# "Rapid Atrial Swirl Sign": A Better Tool Than the Landmark Technique for Ensuring Correct Depth of Insertion of Central Venous Catheters

**DOI:** 10.7759/cureus.65211

**Published:** 2024-07-23

**Authors:** Kairidibo, Arun Raj Pandey, Vandana Dwivedi, Shashi Prakash, Amrita Rath, Reena, Kanak Dwivedi, Ritesh Pandey

**Affiliations:** 1 Anesthesiology and Critical Care, Institute of Medical Sciences, Banaras Hindu University, Varanasi, IND; 2 Pharmacology, Maa Vindhyavasini Autonomous State Medical College (MVASMC), Mirzapur, IND; 3 Pharmacology, Hind Institute of Medical Sciences, Lucknow, IND; 4 Cardiology, Dr. Ram Manohar Lohia Institute of Medical Sciences, Lucknow, IND

**Keywords:** right atrial swirl sign, right atrium, superior vena cava (svc), transthoracic echocardiography (tte), chest-x-ray (cxr), central venous catheter (cvc)

## Abstract

Introduction: Central venous catheters (CVCs) are widely used in the management and resuscitation of critically ill patients in emergency departments and intensive care units. Correct depth of insertion of the CVC line is important to ensure uninterrupted flow, avoid complications, and monitor central venous pressure. Transthoracic echocardiography, with contrast enhancement, has been proposed as an alternative to chest X-ray in detecting central venous line positioning with high accuracy. Nevertheless, this method is not widely used due to some previous conflicting results and the cumbersomeness of the procedure.

Material and methods: After approval by the Institutional Ethics Committee, this prospective observational study was carried out in patients for whom a central venous line was warranted. The study was conducted in the Intensive Care Unit of a tertiary care hospital among 150 adult patients to compare the "Rapid Atrial Swirl Sign" (RASS) technique by transthoracic echocardiography and the landmark-based technique for ensuring accurate depth of central venous line placement.

Results: In this study, we found that the mean depth of insertion of the CVC for the Echocardiography RASS group (E) was 12.84 cm, while for the Landmark technique group (L), it was 12.02 cm. There was a significant difference between these groups, with a p-value of <0.05. We found that the majority of patients (98.63%) in Group E had the catheter tip in Zones 1, 2, and 3, while only 66.6% of patients in Group L had the catheter tip in similar zones. The mean standard deviation for zones on chest X-ray was 1.8 for Group E and 2.26 for Group L, with a significant difference between these groups (p-value <0.05).

Conclusion: The RASS technique is superior to the landmark technique in ensuring the correct depth of the tip of the CVC. When confirmed by chest X-ray, it was found that most patients had the catheter tip in Zone 1, 2, or 3 using the RASS technique. This confirms that the RASS technique can minimize the requirement of resources and hasten the initiation of patient management in a timely manner, unlike the landmark technique, which requires chest X-ray confirmation before use.

## Introduction

The correct depth of insertion of the central venous catheter (CVC) line is crucial to ensure good quality flow, avoid complications such as arrhythmias, pseudoaneurysm, and malpositioning of the catheter, and to measure central venous pressure accurately. Currently, chest X-ray (CXR) is used to ensure the correct depth. However, CXR is not as reliable as "Rapid Atrial Swirl Sign" (RASS) due to individual variations in its interpretation. Additionally, it cannot be repeated frequently and requires extra staff and radiology technician support, whereas bedside echocardiography is readily available in every ICU. The CVC tip should be placed in the last 3 cm of the superior vena cava (SVC) before its junction to the right atrium (SVC-RA) [1]. Mechanical complications of CVC insertions are well known and can be minimized with ultrasound (US) guidance. However, successful venous catheterization does not guarantee optimal positioning of the distal CVC tip, and even with US guidance, there may be concerns about iatrogenic pneumothorax (PTX). CXR use involves exposure to ionizing radiation and requires an available X-ray technician for image acquisition and processing. This may result in a delay in care while the clinician waits to verify CVC positioning by CXR before the catheter can be used. Furthermore, the bedside CXR is usually performed on supine patients, an approach that can lower its accuracy in detecting PTX [2].

Despite its wide use, CXR presents low accuracy when compared to transesophageal echocardiography (TEE), which is considered the gold standard [3]. TEE is the only bedside tool that can directly visualize the CVC tip at the SVC-RA junction, but it is invasive, time-consuming, and requires specific competencies [4]. Transthoracic echocardiography (TTE), with contrast enhancement (CE), has been proposed as an alternative to CXR in detecting CVC positioning with high accuracy. Nevertheless, TTE-CE is not widely used due to some previous conflicting results, and only comparisons between TTE and CXR have been reported [5]. In this study, we aim to use the RASS as a confirmation sign for the correct positioning of the CVC line tip. To confirm the position of the tip, we used CXR as a confirmatory tool. We divided the CXR into five zones based on the distance from the carina, with Zones 1, 2, and 3 being the most appropriate and corresponding to the actual position where a CVC should lie. We compared the RASS technique with the standard landmark technique and confirmed the position of the CVC catheter tip by CXR. This work was also presented as a poster at the national conference of the Indian Society of Critical Care Medicine on February 28, 2024.

## Materials and methods

After the approval of the Institutional Ethical Committee, this prospective observational study was carried out in patients for whom CVC was warranted. The study was conducted in the Intensive Care Unit of a tertiary care hospital in northern India. This study was carried out among 150 adult patients admitted to the ICU from January 2022 to September 2023 to compare the RASS technique by TTE and the landmark-based technique for the correct depth of insertion of the right internal jugular CVC. All patients admitted to the ICU with the need for CVC access who were over 17 years old were included in the study. Patients with a deranged coagulation profile (International Normalized Ratio (INR) > 1.5), those who refused to participate, those with a preexisting internal jugular catheter or indwelling subclavian device, detection of internal jugular thrombosis or right atrial mass, or inability to obtain adequate subcostal or apical four-chamber images were excluded from the study.

Patients were divided into two groups. Group E is the TTE group in which patients underwent right internal jugular triple lumen CVC insertion. Echocardiography (long-axis or four-chamber apical view) was done to assess turbulence around the right atrium and tracheal carina [6]. RASS indicated turbulence upon flushing with 10 ml of normal saline. The absence of swirls suggested malposition. Swirl onset was rated as immediate (<2 seconds), delayed (2-6 seconds), or absent, with different appearances. A score of 3, 2, and 1 was given, respectively, for this. Similarly, based on the intensity of the swirl, it was categorized as prominent, speckling, or absent [7]. Again, a score of 3, 2, and 1 was given, respectively. Ideally, a score of 6 was considered perfect, and the CVC was only fixed once a score of >4 was achieved. A saline flush revealed a swirl that appeared immediately and indicated correct placement. CVC adjustments based on swirl appearance were made, and the catheter was fixed.

Another group was the landmark-based technique group (L). In Group L, the catheter's length was measured naturally over the draped skin from the needle insertion point through the clavicular notch to the second right costal cartilage (tracheal carina level). CVC insertion was guided by US, and the catheter was inserted to the premeasured depth and then fixed in place [8]. CXXRs were taken to confirm its position. Catheter tip locations were assigned based on anatomical zones on the anterior-posterior CXR. The carina serves as an easily identified and reliable anatomic radiographic landmark that correlates well with the midpoint of the SVC [9]. An imaginary line is drawn at the level of the tracheal carina, which is called Zone 1. Zone 2 is considered 3 cm cephalic to the tracheal carina level and to the right of the midline of the chest. This represented the proximal SVC region. Zone 3 is considered 3 cm caudal to the tracheal carina level and to the right of the chest. This represented the distal SVC region. Anywhere above Zone 2 is considered Zone 4, and anywhere below Zone 3 is considered Zone 5. The CVC line between Zones 1 and 3 was considered optimal [10].

In the present study, a minimum of 75 subjects in each group was required to expect similar results with a 10% minimum difference between RASS and the landmark method and to get 80% power and 95% confidence level in the results. Student's t-test and chi-square test were performed to compare the means of both groups. For statistical analysis, IBM SPSS Statistics for Windows, Version 26 (Released 2019; IBM Corp., Armonk, New York) was used. A p-value < 0.05 was considered statistically significant.

## Results

As mentioned in Table 1, our study found that most of the patients who required CVC line insertion were between the ages of 40 and 60 years.

**Table 1:** Distribution of patients according to age Group E: Echocardiography Group; Group L: Landmark Technique Group.

Age Distribution (in Years)	Group E		Group L	
	No. of Patients	Percentage	No. of Patients	Percentage
18-40	31	41.33	16	21.33
41-60	42	56.00	59	78.67
>60	2	2.67	0	0.00
Total	75	100.00	75	100.00
Mean ± SD	44.06±11.92		46.85±11.21	
P-value	0.14			

The male and female preponderance was almost similar for both groups. As mentioned in Table 2, it is very clear that in both groups, males were close to 60%, whereas females were approximately 40%.

**Table 2:** Distribution of patients on the basis of gender Group E: Echocardiography Group; L group: Landmark Technique Group.

Gender	Group E		Group L		P-Value
	No. of Patients	Percentage	No. of Patients	Percentage	
Female	30	40.00	31	41.33	0.86
Male	45	60.00	44	58.67	0.86
Total	75	100.00	75	100.00	

In our study, we found that in Group E, the mean depth of insertion was 12.48 cm, whereas in Group L, it was 12.02 cm (p-value < 0.001), as shown in Table 3.

**Table 3:** Distribution of patients according to the depth of CVC Group E: Echocardiography Group; Group L: Landmark Technique Group, CVC: central venous catheter.

Parameter	Group E		Group L		P-Value
	Mean	SD	Mean	SD	
Depth of CVC (in cm)	12.84	0.94	12.02	0.61	0.001

As shown in Table 4, in 64% of patients in Group E, the swirl appeared in less than 2 seconds, resulting in a score of 3. Similarly, approximately 62.67% of patients had a score of 3 for the intensity of the swirl. In 45.33% of patients, we found a cumulative score (both for time and intensity) of 5. Additionally, 41.33% of patients showed a cumulative score of 6. The mean cumulative score was 5.28.

**Table 4:** Distribution of patients according to cumulative score Group E: Echocardiography Group.

Cumulative Score	Group E	
	No. of Patients	Percentage
4	10	13.33
5	34	45.33
6	31	41.33
Total	75	100.00
Mean ± SD	5.28 0.68	

**Figure 1:**
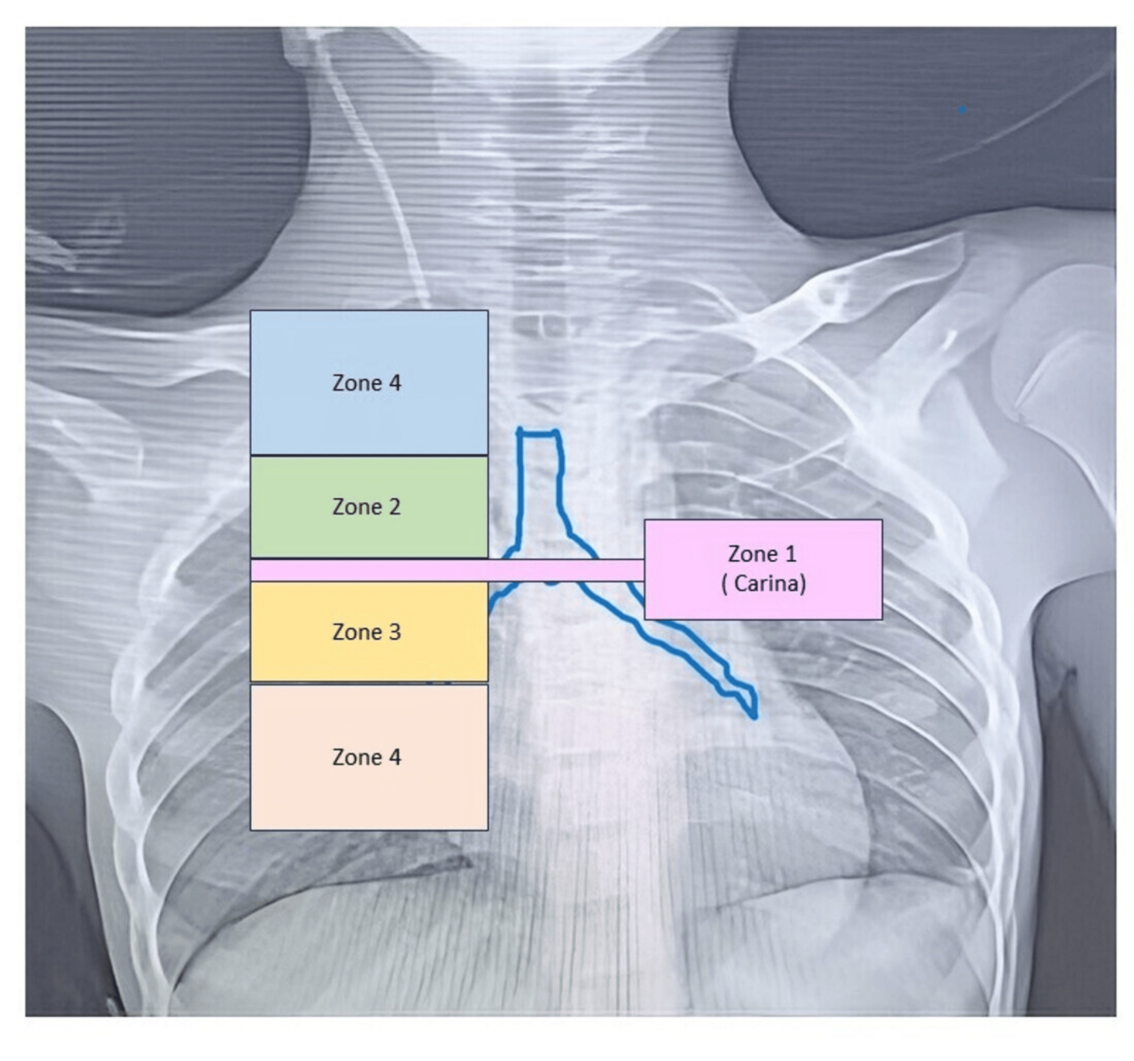
Different zones based on the distance from the carina Zone 1: level at the carina; Zone 3: up to 3 cm below the carina; Zone 2: up to 3 cm above the carina, Zone 4: anywhere above Zone 2; Zone 5: anywhere below Zone 3.

In our study, we divided the CXR into five zones based on the distance from the carina, as shown in Figure 1.

On analysis of the CXRs of both groups, we found that in Group E, approximately 98.63% of CVC lines were present in Zone 1, 2, or 3, whereas in Group L, approximately 66.66% had a CVC line in the same zones. In Group L, 33.33% of patients had a CVC line in Zones 4 and 5, whereas in Group E, the incidence was only 1.33%. As mentioned in Table 5, the mean of zones for Group E was 1.88±0.85, whereas in Group L, it was 2.26±1.10 (mean ± SD), and the difference was statistically significant (p-value < 0.01).

**Table 5:** Distribution of patients according to the zone of chest X-ray Group E: Echocardiography Group; Group L: Landmark-Based Technique Group.

Zone of Chest X-ray	Group E		Group L	
	No. of Patients	Percentage	No. of Patients	Percentage
<4	74	98.63	50	6666
4	1	1.33	25	33.33
Total	75	100.00	75	100.00
Mean ± SD	1.88 ± 0.85		2.26 ± 1.10	
P-value	0.01			

## Discussion

The use and reliance on post-CVC placement CXR entail additional radiation exposure, cost, and resources, with potential time delays to CVC use or clinical management when compared with bedside US. We found that the mean depth of CVC for Group E was 12.84 cm and for Group L it was 12.02 cm. We found a significant difference between these groups, as the p-value was <0.05. Weekes et al. found that the depth of CVC insertion with a triple lumen was 15.2 cm and with Pre Sep it was 17.1 cm [11]. Ahn et al. found that the mean determined depth of catheter insertion was 17.3 (1.2) cm and 16.4 (1.1) cm, respectively, in the Peres formula group and 17.4 (1.2) cm and 16.7 (1.5) cm, respectively, in the Radiological landmark group [12,13]. When compared to our findings, we can conclude that the depth of insertion will be less in the Indian population compared to the above-mentioned study populations. Russell et al. found in their study that for the right side internal jugular vein (IJV) CVC line, 13 cm is sufficient to arrive at the tip at the desired point, which is quite similar to our findings where the mean length of the CVC catheter is 12.84 cm [14].

As all the CVC lines were only fixed once the swirl was seen by saline flush, we did not find a score of 1 in any of our patients. We found that the majority (64%) of patients had a score of three for the time of appearance of the swirl. The mean score was 2.64. The mean score for the intensity of the swirl was 2.62, and the mean cumulative score for Group E was 5.28 [7].

In our study, the majority (98.63%) of patients in Group E had CVC lines located in Zones 1, 2, and 3 on CXR, whereas in Group L, 66.66% of patients had CVC lines in these zones. The mean zone of CXR for Group E was 1.88, and for Group L, it was 2.26. We found a significant difference between these groups, as the p-value was <0.05. Our study, with a specificity of over 98% by the rapid atrial swirl technique, shows that TTE can precisely detect the depth of insertion of CVC in most cases without the need for a CXR. A similar study was done by Korsten et al. in 2018, who found that the precision of insertion depth of the CVC line is very good with RASS assessment using TTE [15].

In a study done in 2018 by Chui et al. on the role of CXR following US-guided CVC insertion, they found that PTX and catheter misplacement after US-guided CVC insertion were rare, and the costs of a post-procedural CXR were exceedingly high [16]. Woodland et al. also concluded that a routine post-procedural CXR is unnecessary and not a wise choice [17]. In our study, we found that US-guided CVC insertion and the detection of the depth of catheter confirmation by 2D echo with a specificity of over 98% indicate that there is no need for a CXR for CVC position confirmation, which also cuts down on cost, time, and resource requirements. Another study can be planned to check the accuracy of RASS as a confirmatory tool for CVC tip position by using TEE or computed tomography. Individual variation in CXR interpretation, the requirement of expertise in echocardiography, and anatomical variation among individuals may be a few of the limitations of this study.

## Conclusions

This study confirms that transthoracic echocardiographic confirmation of CVC position by looking for RASS is a useful method for confirming the depth of insertion and position of the CVC line tip. It has high sensitivity and specificity and can be performed by a person with basic US knowledge. Our study found that the depth of insertion determined by the landmark technique is not as accurate when compared to the RASS technique. Even when using US-guided insertion of the CVC line, the depth of insertion could not be precisely determined with the landmark-based technique. Our study concludes that if the RASS technique is regularly used, it could potentially eliminate the radiation exposure associated with CXR. With the use of this technique, we can also confirm the CVC position in pregnant women and other groups where X-rays cannot be performed. It also confirms that the application of the unconventional RASS technique for CVC position confirmation can minimize the requirement of resources and hasten the process of initiating disease management in a timely manner. This method also rules out the possibility of catheter malposition, ensuring good quality flow and accurate central venous pressure measurement.
